# Blood-saving dissection with monopolar tungsten needle electrodes and Teflon-coated spatula electrodes in tumor orthopedics

**DOI:** 10.1186/s10195-023-00704-8

**Published:** 2023-05-15

**Authors:** Jan Puetzler, Andrea Ulrike Steinbicker, Jana Santel, Niklas Deventer, Michael Jahn, Alexander Zarbock, Georg Gosheger, Martin Schulze, Dana Janina Jenke

**Affiliations:** 1 Department of Orthopaedics and Tumor Orthopaedics, University Hospital Muenster, Albert-Schweitzer-Campus 1, 48149 Muenster, Germany; 2 Department of Anaesthesiology, Intensive Care Medicine and Pain Therapy, University Hospital Frankfurt, Theodor-Stern-Kai 7, 60596, Frankfurt am Main, Germany; 3 Department of Anaesthesiology, Intensive Care and Pain Medicine, University Hospital Muenster, Albert- Schweitzer-Campus 1, 48149, Muenster, Germany

**Keywords:** Electrosurgery, Blood loss, Patient blood management, Tumor orthopedics, Tumor endoprosthesis

## Abstract

**Introduction:**

Resection of musculoskeletal tumors and reconstruction with tumor endoprostheses often results in blood loss requiring transfusion of blood products. We assessed the blood-saving potential of using monopolar tungsten needle electrodes and polytetrafluoroethylene (PTFE)-coated spatula electrodes (intervention) compared with conventional dissection with sharp instruments and coagulation with uncoated steel electrodes (control).

**Methods:**

We retrospectively analyzed data of 132 patients (79 interventions, 53 controls) undergoing surgery by one single experienced surgeon in our tertiary referral center between 2012 and 2021.

**Results:**

Intraoperative blood loss in the intervention group was reduced by 29% [median (IQR): 700 (400–1200) vs 500 (200–700) ml; *p* = 0.0043]. Postoperative wound drainage decreased by 41% [median (IQR): 1230 (668–2041) vs 730 (450–1354) ml; *p* = 0.0080]. Additionally, patients in need of PRBCs during surgery declined from 43% to 15% (23/53 vs 12/79; *p* = 0.0005), while the transfusion rate after surgery did not change notably. The number of patients in need of revision surgery due to wound healing disorders was low in both groups (control group: 4/53 vs intervention group: 4/79). Only one patient in the control group and two patients in the intervention group underwent revision surgery due to hemorrhage. Baseline characteristics were similar between groups (sex, Charlson Comorbidity score, tumor entity).

**Conclusion:**

Dissection with tungsten needle electrodes and PTFE-coated spatula electrodes appears an effective surgical blood-saving measure without increased risk of wound healing disorders.

***Level of evidence*:**

III, retrospective comparative study.

*Clinical trial registration*. The study was registered at ClinicalTrials.gov. Identifier: NCT05164809.

## Introduction

Extremity tumor resection and reconstruction with tumor endoprostheses can result in relevant blood loss that requires hemodynamic stabilization and transfusion of blood products. This is mainly because extra-anatomical approaches are necessary to achieve a wide resection according to Enneking, involving large wound cavities and exposure of large tumor-supplying blood vessels [1]. While in several other surgical fields machine autotransfusion is an intraoperative blood-saving method, this is avoided in tumor surgery as it bears the risk of hematogeneous dissemination of tumor cells [2, 3]. Therefore, additional surgical measures are needed to reduce blood loss in tumor orthopedics.

A feasible approach seems to be the use of monopolar electrosurgery in order to improve hemostasis during tumor dissection. At temperatures over 100 °C tissue fluid vaporizes abruptly, proteins are denatured, blood vessels shrink, and opposing vessel walls are thermally fused, thus achieving hemostasis while performing an incision. Minimizing heat damage to the tissue adjacent to the dissection is achieved by keeping the diameter of the working electrode as small as possible. Electrical energy is concentrated at the narrowest part of the electrode; thus, less power is required to maintain the cutting effect without encountering high tissue resistance. Needle electrodes made of tungsten are particularly suitable for this purpose as tungsten has the highest melting point of all metals (at 3422 °C); therefore it does not deform, and is highly abrasion resistant. In 1994, Peterson observed reduced blood loss with a tungsten needle electrode for scalp reduction surgery, although he did not quantify this observation [4]. The electric properties of tungsten resulted in less tissue damage in endoscopic polypectomy in an in-vivo pig study compared with steel electrodes [5]. Spatula electrodes with polytetrafluoroethylene (PTFE) coating can be used for dissection of deep tissues with high efficiency in coagulating larger vessels. They create less eschar and smoke compared with uncoated stainless-steel electrodes.

To our knowledge, no clinical study has yet investigated the benefit of the combined use of these monopolar electrodes in a surgical field that, due to the inherent conditions, causes a relatively high blood loss and transfusion requirement. With this study, we assess whether dissection with these electrodes could reduce intraoperative blood loss, postoperative wound drainage, and the need for transfusions compared with conventional dissection without these electrodes. In addition, the potential risk for revision surgery due to wound healing disorders, as described in early animal studies, was evaluated [6].

## Methods

This is a single-surgeon retrospective cohort study based on the recorded data of 132 patients treated in our tertiary referral center between 2012 and 2021. All procedures in this study were performed in accordance with the ethical standards of the institutional research committee and with the 1964 Helsinki Declaration and its later amendments. Ethical approval was obtained from our local ethics committee (approval number: 2021-527-f-S).

**Fig. 1:**
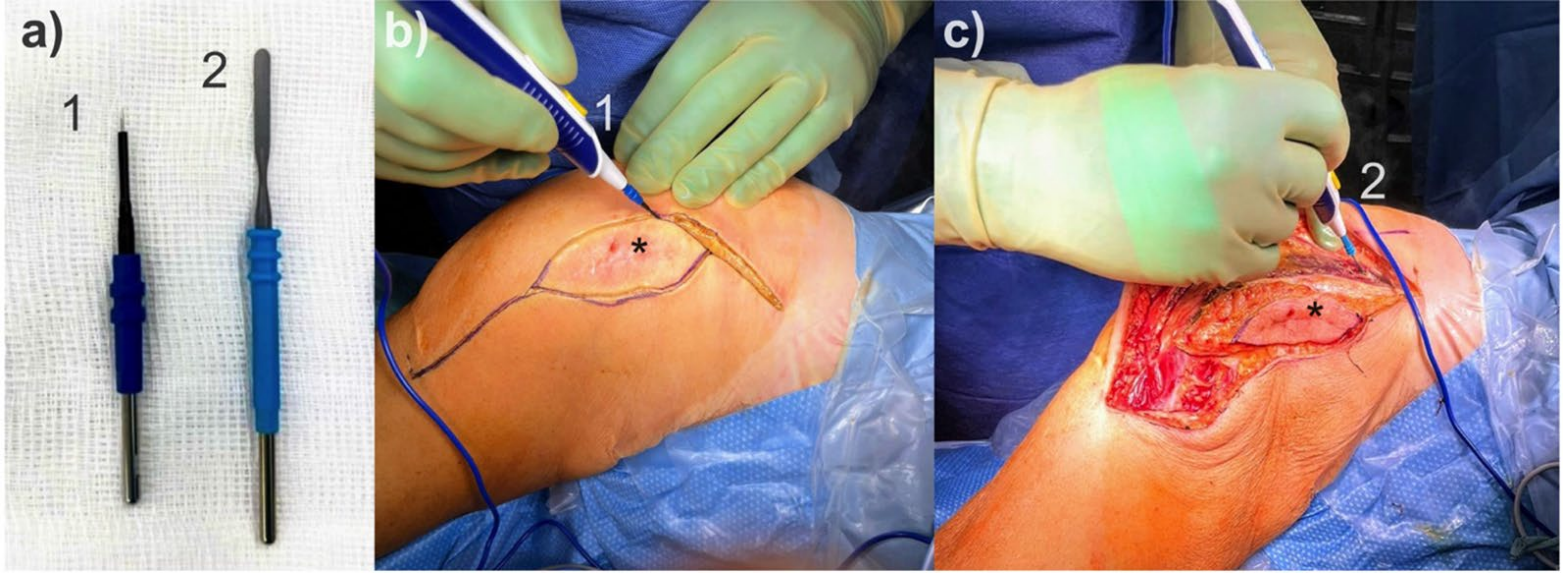
Intraoperative photographs of tumor dissection performed exclusively with monopolar electrodes; **a** Monopolar tungsten needle electrode (*1*), Teflon-coated spatula electrode (*2*) (both: Erbe Elektromedizin GmbH, Tuebingen, Germany). **b** Skin incision and superficial dissection of the biopsy channel (*), that is inevitably contaminated with tumor cells after incisional biopsy and thus must be completely removed together with the tumor. This step is performed with the high-precision tungsten needle electrode (*1*). **c** Deep dissection to achieve wide tumor resetion is then performed with the teflon-coated spatula electrode (*2*). Visibility of the surgical site is excellent due to minimal bleeding

The combined use of tungsten needle electrodes and PTFE-coated spatula electrodes for dissection of musculoskeletal tumors, as shown in Fig. 1, has not yet been described in the literature. This technique was introduced at our hospital in 2017 (tungsten needle electrode REF 21191-316 and PTFE-coated spatula electrode REF 21191-459, Electrosurgical unit: VIO 300D; Erbe Elektromedizin GmbH, Tuebingen, Germany). Both electrodes are commercially available in over 100 countries (as of January 2023). The settings for the tungsten needle electrode are: AUTO CUT Mode, maximum power output at rated load resistor: 100 W ± 20%, Effect: 1. This electrode is used for skin incisions and superficial subcutaneous tissue. Then it is changed to the PTFE-coated spatula electrode for deep dissection. The corresponding settings are: TWIN COAG Mode, maximum power output at rated load resistor: 200 W ± 20%, Effect: 5. These electrodes have been used for dissection in our practice since 2017 as a standard method (intervention group). Previously, disposable scalpels were used for skin incision and deep dissection was performed with sharp instruments and uncoated stainless-steel electrodes for hemostasis (control group).

After tumor resection and reconstruction with tumor endoprostheses a minimum of one subfascial and one subcutaneous drainage were inserted as a standard protocol for all studied patients. These drainages were left in place until wound secretion was less than 50 ml per 24 h, or the maximum duration of 5 days was reached. All tumor endoprostheses that were implanted belonged to a single design implant system (modular tumor and revision system, MUTARS, Implantcast GmbH, Buxtehude, Germany).

**Fig. 2:**
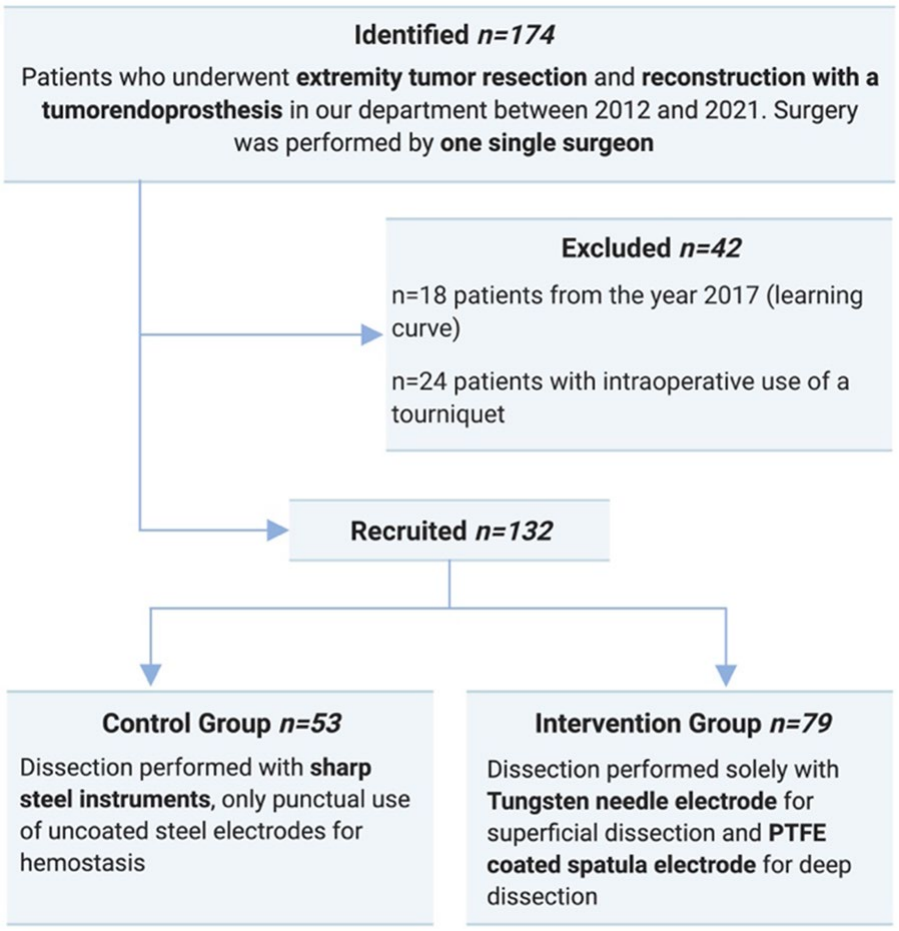
Flow of participants included in the analysis

We identified 174 patients who underwent tumor resection and reconstruction with tumor endoprostheses by one single surgeon between 2012 and 2021. This surgeon had over 20 years of experience in this field in 2012 when the observation period began. This should minimize potential bias of a learning curve. All surgeries in 2017 were excluded (*n* = 18) as this year was regarded a transitional period from the old technique to the new electrodes. With the novel electrodes, the need for tourniquets diminished to provide a bloodless surgical field. We excluded all 24 cases where a tourniquet was used from our cohort to adjust for this potential confounder. This resulted in a total of 132 patients that were included in the final analysis (*n* = 53 in the control group and *n* = 79 in the intervention group; Fig. 2).

### Description of outcomes

Intraoperative blood loss was assessed via the volume in the suction collection containers and documented in the anesthesia protocol in a free text-box. The volume of rinsing liquid during surgery was deducted. Postoperative wound drainage was assessed via the volume in collection containers from suction tubes and documented twice daily by nurses in the digital patient chart in a free text-box. The amount of transfused packed red blood cells (PRBCs) in units was documented intraoperatively in the anesthesia protocol and postoperatively in the digital patient chart by the treating physicians. One unit comprised 250–350 ml of allogenic concentrated erythrocytes.

Additional data of the patients were retrieved from the digital patient chart including age, sex, comorbidities, tumor entity, surgery time, anticoagulants, coagulation parameters [international normalized ratio (INR), partial thromboplastin time (pTT)], hemoglobin level and hematocrit (value before surgery and lowest value within 7 days after surgery), duration of hospitalization in days, creatinine level (value before surgery and highest value within 7 days after surgery), renal insufficiency requiring dialysis pre- and post-surgery during hospitalization, and number of revision surgeries needed due to impaired wound healing or postoperative bleeding.

### Statistical analysis

Statistical analyses were performed using GraphPad Prism version 9.4.0 for macOS (GraphPad Software, San Diego, California USA). All *p*-values and confidence limits were two-sided and intended to be exploratory, not confirmatory. Therefore, no adjustment for multiplicity was made. Two-sided *p*-values ≤ 0.05 were considered statistically significant.

In the descriptive analysis, continuous variables are reported as median (25% quantile–75% quantile, IQR). Absolute and relative frequencies are given for categorical variables. Groups were compared using the Mann–Whitney *U*-test or Student’s *t*-test, depending on normality, for continuous data; and Fisher’s exact test for categorical variables. The Shapiro–Wilk test was used to assess normality.

## Results

### Baseline characteristics

Baseline characteristics, tumor entity and tumor location were similar in both groups, except for higher age (Table 1) and a larger proportion of metastases in the intervention group (Table 2).

**Table 1:** Overview of patient characteristics in the two study groups

Patient characteristics	Control group (*n* = 53)	Intervention group (*n* = 79)	*p*-value
Age in years, median (IQR)	29 (12.5–55)	48 (16–69)	0.0435^*^
Female, *n* (%)	19 (36%)	40 (51%)	0.1098^†^
Male, *n* (%)	34 (64%)	39 (49%)	0.1098^†^
Charlson comorbidity score, median (IQR)	2 (2–6)	3 (2–7)	0.1388^*^

*IQR* interquartile range

Bold font indicates *p*-value < 0.05

^*^p-value from Mann–Whitney *U*-test

^†^*p*-value from Fischer’s exact test

**Table 2:** Tumor entities and location in the two study groups

Tumor entity	Control group (n = 53)	Intervention group (n = 79)
Osteosarcoma, *n* (%)	25 (47%)	28 (35%)
Metastasis, *n* (%)	4 (8%)	21 (27%)
Ewing sarcoma, *n* (%)	10 (19%)	12 (15%)
Chondrosarcoma, *n* (%)	8 (15%)	7 (9%)
Myxofibrosarcoma, *n* (%)	0 (0%)	3 (4%)
Giant cell tumor, *n* (%)	2 (4%)	2 (3%)
Other, *n* (%)	4 (8%)	6 (8%)
Tumor location		
Upper arm		
Proximal upper arm, *n* (%)	12 (22.6%)	6 (7.6%)
Distal upper arm, *n* (%)	0 (0%)	1 (1.3%)
Total upper arm, *n* (%)	1 (1.9%)	0 (0%)
Forearm		
Proximal forearm, *n* (%)	1 (1.9%)	0 (0%)
Distal forearm, *n* (%)	0 (0%)	0 (0%)
Thigh		
Proximal thigh, *n* (%)	17 (32.0%)	20 (25.3%)
Distal thigh, *n* (%)	14 (26.4%)	27 (34.2%)
Total thigh, *n* (%)	5 (9.4%)	9 (11.4%)
Total knee, *n* (%)	0 (0%)	2 (2.5%)
Lower leg		
Proximal lower leg, *n *(%)	2 (3.8%)	13 (16.5%)
Distal lower leg, *n* (%)	1 (1.9%)	1 (1.3%)

### Outcomes: intraoperative blood loss, postoperative wound drainage, transfusions

**Fig. 3:**
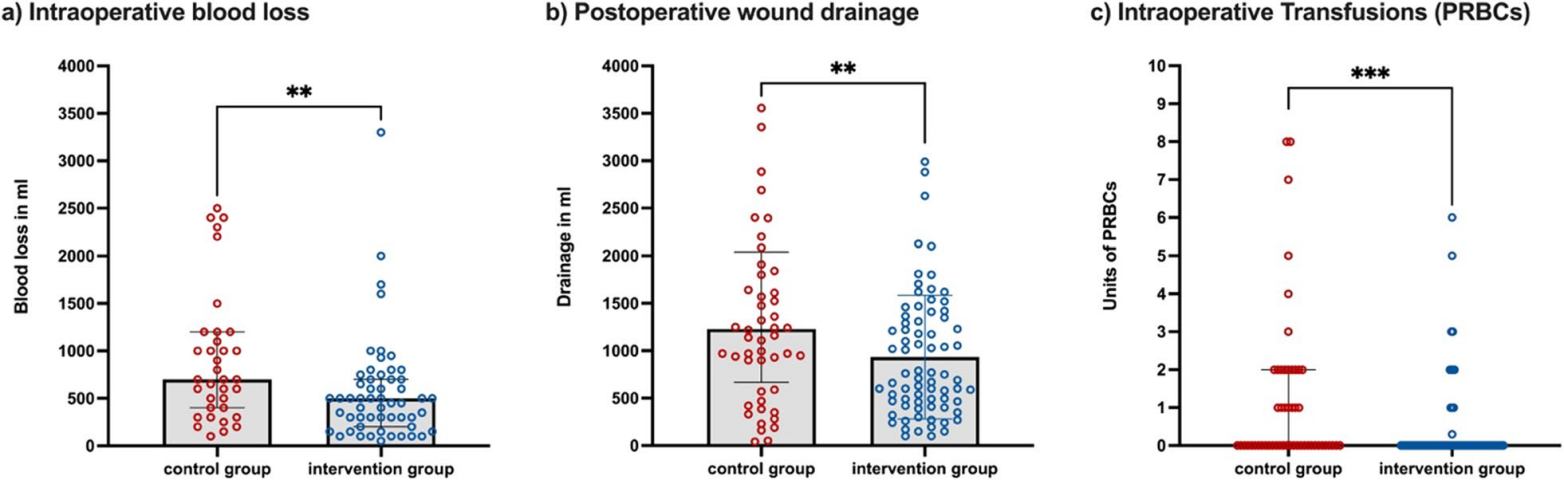
Comparison of main outcomes between the intervention group and the control group. **a** Intraoperative blood loss in ml. **b** Postoperative wound drainage in ml. **c** Intraoperative transfusions of packed red blood cells (PRBCs) in units. *Bars* indicate median, *error bars* indicate interquartile range, ** *p*-value <0.01, *** *p*-value <0.001

Blood loss in the intervention group was reduced by 29% [intervention group: median (IQR): 700 (400–1200) vs control group: 500 (200–700) ml; *p* = 0.0043] (Fig. 3a, Table 3).

**Table 3:** Overview of the primary outcome parameters of the two study groups

Primary outcome parameters	Control group (*n* = 53)	Intervention group (n = 79)	*p*-value
Intraoperative blood loss (ml), median (IQR)	700 (400–1200)	500 (200–700)	0.0043^*^
Postoperative wound drainage (ml), median (IQR)	1230 (668–2041)	730 (450–1354)	0.0080^*^
Intraoperative transfusion of PRBCs (units)			
Median (minimum–maximum)	0 (0–22)	0 (0–6)	0.0003^*^
0 units, *n* (%)	30 (57%)	68 (86%)	**0.0023**^†^
1 unit, *n* (%)	7 (13%)	3 (4%)	
2 units, *n* (%)	9 (17%)	4 (5%)	
> 2 units, *n* (%)	7 (13%)	4 (5%)	
Postoperative transfusions of PRBCs (up to 14 days; units)			
Median (minimum–maximum)	0 (0–6)	0 (0–4)	0.1541^*^
0 units, *n* (%)	35 (66%)	61 (77%)	0.4781^†^
1 unit, *n* (%)	5 (9%)	6 (8%)	
2 units, *n* (%)	8 (15%)	6 (8%)	
> 2 units, *n* (%)	5 (9%)	6 (8%)	

*IQR* interquartile range, *PRBCs* packed red blood cells

Bold font indicates *p*-value < 0.05

^*^p-value from Mann–Whitney *U*-test

^†^*p*-value from Fischer’s exact test

Postoperative wound drainage assessed via suction tubes was decreased by 41% [intervention group: median (IQR): 1230 (668–2041) vs control group: 730 (450–1354) ml; *p* = 0.0043] (Fig. 3b, Table 3). The amount of units of PRBCs given intraoperatively were reduced in the intervention group (Fig. 3c, Table 3).

**Fig. 4:**
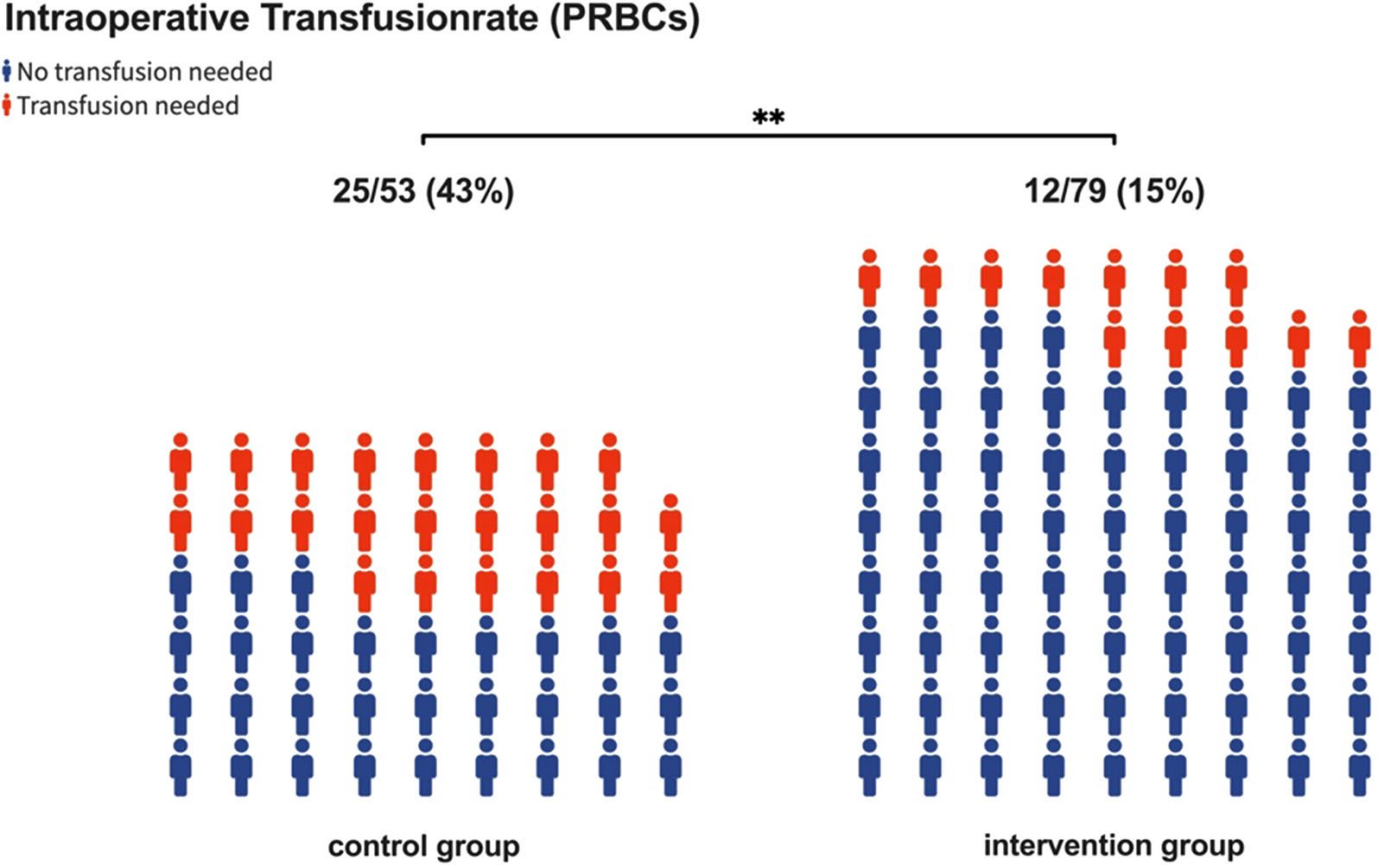
Comparison of the number of patients in need of transfusions with packed red blood cells (PRBCs) during surgery between the intervention group and the control group. ** *p*-value <0.01

In addition, the intraoperative transfusion rate (number of patients in need of transfusions) was reduced from 43% in the control group to 15% in the intervention group (23/53 vs 12/79; *p* = 0.0005) (Fig. 4).

No notable difference was observed for postoperative PRBC transfusions within 14 days after surgery (Table 3). The postoperative transfusion rate between the two groups with 34% in the control group and 23% in the intervention group did not differ significantly (18/53 vs 18/79; *p* = 0.17).

Similar results were observed regarding the secondary outcome parameters: surgical time, number of surgical revisions, and hospitalization time (Table 4).

**Table 4:** Overview of the secondary outcome parameters of the two study groups

Secondary outcome parameters	Control group (n = 53)	Intervention group (n = 79)	*p*-value
Surgical time [incision to suture time in minutes, median (IQR)]	201 (157–272)	185 (145–243)	0.1563^*^
Length of hospital stay [days, median (IQR)]	18 (11–27)	14 (10–27)	0.1744^*^
Patients needing revision surgery due to wound healing disorders, *n* (%)	4 (8%)	4 (5%)	0.7133^†^
Patients needing revision surgery due to hemorrhage, *n* (%)	1 (2%)	2 (2.5%)	> 0.9999^†^

*IQR* interquartile range

^*^p-value from Mann–Whitney *U*-test

^†^p-value from Fischer’s exact test

Laboratory parameters between the two groups did not differ in terms of hemoglobin, hematocrit, creatinine, and the international normalized ratio (INR) (Table 5). The intake of acetylsalicylic acid 100 mg per day as permanent medication was similarly rare in both groups (8/79 vs 2/53; *p* = 0.3143).

**Table 5:** Overview of laboratory parameters of the two study groups

Laboratory parameters	Control group (*n* = 53)	Intervention group (*n* = 79)	*p*-value
INR preop., median (IQR)	1.04 (0.97–1.09)	1.02 (0.97–1.09)	0.5432^*^
Hemoglobin difference (preop. − postop^a^) (g/dl)	3.6 (1.9–4.7)	3.3 (2.4–4.7)	0.8143^*^
Hematocrit difference (preop. − postop^a^) (%)	10.7 (6.6–14.8)	10.4 (7.4–14.2)	0.7191^*^
Creatinin difference (preop. − postop^a^) (mg/dl)	0.1 (− 0.1 to 0.2)	0.0 (− 0.1 to 0.1)	0.2555^*^

*IQR* interquartile range, *INR* International standardized ratio, *pTT* partial thromboplastin time

Bold font indicates *p*-value < 0.05

^*^p-value from Mann–Whitney *U*-test

^a^Subtraction of postoperative value minus preoperative value. Preoperative value: last value before surgery (not older than 7 days). Postoperative value: highest value (creatinin) or lowest value (hemoglobin, hematocrit) within 7 days after surgery

No patient suffered from acute kidney injury postoperatively. One patient in the control group was already in a dialysis-dependent state prior to surgery due to chronic renal failure.

## Discussion

### Reduction of blood loss

We observed a relevant reduction of intraoperative blood loss in the intervention group using monopolar tungsten needle electrodes and PTFE-coated spatula electrodes for tumor dissection. In the literature for orthopedic surgery, only two previous studies evaluated the effect of electrosurgical dissection as a blood-saving measure. Widman et al*.* in 1999 found no significant difference in total blood loss in 67 patients with primary hip arthroplasty [7]. In 33 patients the scalpel was used throughout the operation and diathermy only for coagulation of bleeding spots. In 34 patients a diathermy knife was used solely. No wound infections or relevant postoperative bleeding occurred in either group.

Byrne et al*.* in 2007 found a reduction of blood loss in relation to the wound size using electrosurgery in hemiarthroplasty in 100 patients with hip fractures operated on by one single surgeon [8]. Bleeding from bone marrow during reaming was considered a confounder, reducing the overall effect of electrosurgery on total blood loss in their study. In our study, the blood-saving effect was still very pronounced, although we did not differentiate between blood loss during soft tissue dissection and the following reconstruction phase.

### Reduction of intraoperative transfusions of PRBCs

In total, fewer PRBCs were transfused and a lower number of patients received transfusions in the intervention group during surgery. To date, there are only a few reports of surgical liver resections in which the use of monopolar electrodes has resulted in a reduction of the number of required transfusions of PRBCs [9]. So far, no comparable results have been published in the current orthopedic literature.

### Reduction of wound drainage

A significant reduction in postoperative wound drainage was observed in the intervention group. This drainage fluid consists of portions of blood and wound exudate released over the wound surfaces. Persistent wound secretion over several days may cause delayed wound healing or even promote development of infection, and may require surgical revision. The combination of cutting and simultaneous sealing of small blood and lymphatic vessels during dissection might have created a dry wound that resulted in reduced wound discharge. In this instance, our results contradict earlier studies that found larger formation of postoperative seroma when electrosurgery was used. A meta-analysis of Ismail et al*.*, including eleven studies (1258 participants), reported higher rates of seroma formation in the diathermy group [10]. The authors suggested that heat damage to adjacent tissues resulted in inflammation, promoting excessive wound discharge and seroma formation. Higher cytokine levels in drainage fluids after mastectomy indicated that electrosurgery could in fact induce a stronger acute inflammatory response compared with scalpel dissection [11]. Whether the use of PTFE-coated electrodes and tungsten needle electrodes used in our study, powered by a modern oscillator unit that can deliver pure sinusoidal current, may cause less thermal damage than earlier diathermy electrodes reported in the literature cannot be conclusively determined here.

### Wound healing disorders and postoperative hemorrhage

The overall rate of wound healing disorders and postoperative hemorrhage needing surgical revision in our study is in keeping with reports in the literature [12, 13]. These complications occurred with comparable frequency in the control and the intervention group. Ismail et al*.* also found no difference in wound healing and infections, length of hospital stay, and scar formation in their meta-analysis [10]. They included 41 studies with 6422 participants, but they were mainly abdominal surgeries, and there were only two studies in the orthopedic field [7, 8]. Early animal studies from 1980 raised concerns about potential increased wound healing disorders and infections when diathermy was used for skin incisions [6] and the National Institute for Health and Clinical Excellence (NICE) guidelines in 2008 objected to the use of diathermy for skin incisions [14]. This recommendation has not been revisited in the current 2019 edition [15]. Nevertheless, clinical trials, including our study, have not found an association between wound healing disorders and monopolar electrosurgery [16, 17].

### Limitations

To our knowledge, this is the first study to evaluate the use of these novel electrodes regarding blood-saving potential in tumor orthopedics. However, several limitations need to be considered when interpreting these results. This is a retrospective analysis with a historical control group, which has an inherent risk of bias, as other conditions and parameters may have changed over time, affecting our parameters of interest. In order to achieve a high level of standardization between the groups we limited the inclusion criteria to patients that were operated on by one single surgeon who already had 20 years of experience in this field at the beginning of the observation period. So, the potential bias due to a learning curve or varying skills of different surgeons was eliminated. We assume that after 20 years of experience in tumour orthopedics, surgical skills have matured to a level where an additional 9 years might not significantly influence the outcomes of interest.

Furthermore, the intervention group was on average 9 years older. An explanation could be that older patients are increasingly considered fit for surgery. Chronological age is no longer regarded to be contraindication in attempting curative surgical procedures in oncological surgery [18]. Age-matched groups would be desirable to investigate our hypothesis in conditions that are as standardized as possible, but would further reduce the number of patients available for analysis. We proceeded from the assumption that higher chronological age in adults does not itself contribute to a reduction in blood loss and have therefore refrained from age adjustment.

The principles of patient blood management were already introduced in our hospital at the beginning of our observation period in 2012, but an influence on the reduction of intraoperative transfusions by more restrictive transfusion triggers, which were increasingly applied during our study, cannot be completely excluded [19]. However, blood loss during surgery is a parameter that is not affected by this. In addition, the postoperative transfusion rate after surgery and the hemoglobin levels after surgery were similar in both groups. This suggests that the criteria for administering transfusions did not change significantly during the observation period, otherwise lower hemoglobin levels should be observed in the intervention group. Due to the retrospective design, this is not a confirmatory, but an exploratory approach, and the observed results would need to be confirmed in prospective studies.

## Conclusion

Monopolar electrosurgical dissection with tungsten needle electrodes and PTFE-coated spatula electrodes appears a relevant contribution to the concept of patient blood management in tumor orthopedics.

## Data Availability

The raw datasets are available from the corresponding author on reasonable request.
